# Synthetic Physical Interactions Map Kinetochore-Checkpoint Activation Regions

**DOI:** 10.1534/g3.116.031930

**Published:** 2016-06-08

**Authors:** Guðjón Ólafsson, Peter H. Thorpe

**Affiliations:** The Francis Crick Institute, Mill Hill Laboratory, London, NW7 1AA, United Kingdom

**Keywords:** kinetochore, Mad1, Mad2, Mps1, SAC

## Abstract

The spindle assembly checkpoint (SAC) is a key mechanism to regulate the timing of mitosis and ensure that chromosomes are correctly segregated to daughter cells. The recruitment of the Mad1 and Mad2 proteins to the kinetochore is normally necessary for SAC activation. This recruitment is coordinated by the SAC kinase Mps1, which phosphorylates residues at the kinetochore to facilitate binding of Bub1, Bub3, Mad1, and Mad2. There is evidence that the essential function of Mps1 is to direct recruitment of Mad1/2. To test this model, we have systematically recruited Mad1, Mad2, and Mps1 to most proteins in the yeast kinetochore, and find that, while Mps1 is sufficient for checkpoint activation, recruitment of either Mad1 or Mad2 is not. These data indicate an important role for Mps1 phosphorylation in SAC activation, beyond the direct recruitment of Mad1 and Mad2.

To maintain the correct complement of chromosomes during cell division, accurate segregation of chromosomes is essential. The spindle assembly checkpoint (SAC) is a key mechanism that regulates this process, and prevents cells becoming aneuploid by inhibiting the anaphase promoting complex/cyclosome (APC/C) (Musacchio and Salmon 2007; Jia *et al.* 2013). The SAC is activated by the sequential recruitment of checkpoint proteins to the kinetochore—the large protein assembly that links the centromere to the spindle microtubules (Foley and Kapoor 2013; Jia *et al.* 2013). First, Bub1 and Bub3 are recruited to the kinetochore in response to phosphorylation of Spc105 (KNL1) “MELT”-like motifs by the Mps1 kinase (London *et al.* 2012; Shepperd *et al.* 2012; Yamagishi *et al.* 2012; Primorac *et al.* 2013). Phosphorylated Bub1 recruits Mad1 (London and Biggins 2014), which in turn recruits Mad2. This Mad1–Mad2 complex then templates the conversion of an ‘open’ form of Mad2 (O-Mad2) to a ‘closed’ form (C-Mad2) (Luo *et al.* 2004; De Antoni *et al.* 2005). The C-Mad2 isoform, together with Mad3 and Bub3, forms the mitotic checkpoint complex (MCC) with Cdc20, preventing the activity of APC/C^Cdc20^ (Sudakin *et al.* 2001; De Antoni *et al.* 2005; Herzog *et al.* 2009; Stukenberg and Burke 2009; Barford 2011; Han *et al.* 2013). A key assumption is that the kinetochore enrichment of these checkpoint proteins is necessary, and, in some cases, sufficient, for the activation of the SAC. This notion has been tested by artificially recruiting individual checkpoint proteins to the kinetochore and associated proteins (Maldonado and Kapoor 2011; Ito *et al.* 2012; Lau and Murray 2012; Ballister *et al.* 2014; Kruse *et al.* 2014; London and Biggins 2014; Aravamudhan *et al.* 2015); however, the systematic recruitment of checkpoint proteins to each kinetochore protein has not been examined. To ask whether recruitment of checkpoint proteins *per se* is sufficient for checkpoint activation, we tethered Mad2 to most other proteins in the cell. Although we identify a number of proteins that, when associated with Mad2, block cell cycle progression, surprisingly we could not activate the SAC directly by tethering Mad2, even a constitutively active form of Mad2, to the kinetochore. The only exception is the association of Mad2 with Cse4, which robustly activates the SAC. However, we show that this interaction likely acts indirectly, first disrupting kinetochore structure, which then activates the SAC. We then associated Mad1 with the kinetochore proteins and, as for Mad2, we could not produce SAC-dependent arrest. However, we do show that Mad1 tethered to the kinetochore can participate in a checkpoint response. In contrast, recruitment of the upstream checkpoint kinase, Mps1, produced robust cell cycle arrest when recruited to the KMN (KNL1/MIND/Ndc80) network of proteins, and this arrest was dependent upon the kinase activity of Mps1 and downstream checkpoint components. Our data suggest that locally high concentrations of Mad1 and Mad2 at the yeast kinetochore are not, alone, sufficient for checkpoint activation.

## Materials and Methods

### Yeast methods

The yeast strains used in this study are listed in Supplemental Material, Table S2. Strains were constructed using standard techniques, and standard yeast growth medium including 2% (w/v) of the indicated carbon source (Sherman 2002). Yeast plasmids were created using the gap-repair cloning technique, which combines a linearized plasmid with PCR products using *in vivo* recombination. All PCR products were generated using primers from Sigma Life Science and PfuII Ultra proof reading polymerase (Agilent Technologies, UK). All plasmid constructs (listed in Table S3) were validated using Sanger sequencing (Beckman Coulter Genomics, UK). Selective ploidy ablation (SPA) screening followed the established protocol using the donor strain W8164-2B (Reid *et al.* 2011), and utilizing a ROTOR pinning robot (Singer Instruments, UK).

### Synthetic physical interaction screens

Synthetic physical interaction technology (SPI) screens were performed as previously reported (Ólafsson and Thorpe 2015). The GBP alleles were expressed ectopically in GFP strains from a single-copy plasmid under the control of a *CUP1* promoter; all strains were grown at 30°. Plasmids encoding the GBP-tagged proteins and controls were transferred separately into the GFP strains using SPA (Reid *et al.* 2011). Arrays of 96 or 384 strains from the GFP collection were grown on rectangular plates containing YPD medium, typically at 1536 colonies per plate density (16 or four replicates of each strain, respectively). After growth overnight, these *MAT***a** plates were copied onto new YPD plates with a lawn of donor *MATα* yeast strain containing a specific *LEU2* plasmid. The resulting cells were grown overnight on YPD to allow mating, and then copied onto medium containing galactose and lacking leucine (GAL –leu) to compromise the conditional chromosomes of the donor strain, while selecting for the specific plasmid. After 24 hr the colonies were replicated to GAL –leu medium containing 5-fluoroorotic acid (GAL –leu 5FOA) to select against the remaining donor chromosomes, and 24–48 hr later the plates with resulting haploids containing the plasmid were imaged using a desktop scanner in transmission mode (minimum resolution 300 dpi). Quantitative analysis of the resulting colony size was performed as previously reported (Ólafsson and Thorpe 2015). First, the colony sizes on each plate were normalized to a median value of 1 to account for plate to plate variations in growth. Next, for each strain, the average colony size of the control was compared with that of the experimental plasmid(s), to generate a growth ratio (a growth ratio of 1 indicates equal colony sizes on control *vs.* experiment; higher growth ratios indicate that experimental colonies are smaller than controls). The natural log of the growth ratios are reported here for each strain; LGR = ln(control size/experimental size). To put this in context, an LGR of 1 equates to an experimental colony 37% of the size of a control colony, and an LGR of 0.4 equates to an experimental colony 67% of the size of a control colony.

### Fluorescence microscopy

To examine the location of tagged proteins within cells, we used epifluorescence microscopy. Log phase cells were embedded in 0.7% low melting point agarose dissolved in the appropriate growth medium. The depth of agarose between the slide and coverslip is fixed at 6–8 µm, slightly larger than the diameter of the average yeast cell, which maintains a consistent distance from the coverslip to the cell nucleus. Cells were imaged with a Zeiss Axioimager Z2 microscope (Carl Zeiss AG, Germany), using a 63 × 1.4NA oil immersion lens, illuminated using a Zeiss Colibri LED illumination system (CFP = 445 nm, GFP = 470 nm, YFP = 505 nm, RFP = 590 nm). Bright field contrast was enhanced with differential interference contrast (DIC) prisms. The resulting light was captured using a Hamamatsu ORCA ERII CCD camera containing an ER-150 interline CCD sensor with 6.45 µm pixels, binned 2 × 2, or a Hamamatsu Flash 4.0 Lte. camera containing a FL-400 CMOS sensor with 6.5 µm pixels, binned 2 × 2 (Hamamatsu Photonics, Japan). The exposure times were set to ensure that CCD pixels were not saturated. The ORCA ERII 12 bit images have a pixel size of 205 nm in x and y, a z step size of 300 nm, and an effective dynamic range of ∼3000 gray levels. The Flash 4.0 16-bit images have a pixel size of 206 nm in x and y, a z step size of 300 nm, and an effective dynamic range of ∼30,000 gray levels. Images shown in the figures were prepared using Volocity imaging software (Perkin Elmer Inc., USA). For merged images of different fluorescent channels, the contrast was adjusted in a linear way to ensure that the background appears black.

### Fluorescence image analysis

A quantitative measurement of Smc1-YFP fluorescence (Figure S3, C and D) was measured using a custom script for Volocity software. Briefly, the CFP signal produced from a tagged histone was used to select 3D regions (volumes) representing the nucleus. The mean YFP intensity (Smc1-YFP) within this volume was measured. To correct for background, an external volume three pixels from the edge of the CFP-defined nucleus was chosen, and the mean YFP intensity in this background region was subtracted from the nuclear signal to produce a background-corrected value for each nucleus measured.

### Plasmid loss assay

To assess plasmid loss of cells expressing either of the fusions *MAD2-CSE4* or *CSE4-MAD2*, or, as controls, *MAD2* or *CSE4* alone, along with an empty plasmid containing a NAT selection marker, were grown overnight in –leu +NAT medium, and then switched to GAL –leu for 7 hr to express the *MAD2-CSE4* or *CSE4-MAD2* fusions. For at least eight replicates, ∼500–1000 cells with each of the four expression plasmids were then plated out on –leu and –leu NAT plates to assess the frequency of plasmid loss.

### Chromosome loss assay

To assess chromosome loss, strains carrying an artificial chromosome containing *SUP11*::*URA3* (Spencer *et al.* 1990) and the *MAD2-CSE4*, *CSE4-MAD2*, *MAD2* or *CSE4* plasmids were grown overnight in –leu –ura medium, and the cultures were then spun down and washed before splitting them between galactose –leu –ura medium to express the fusions, or glucose –leu –ura medium to repress expression, for 7 hr. Then a colony sectoring assay was performed by plating out ∼1000 cells on YPD plates; resulting red sectored colonies were scored as chromosome loss events.

### Data availability

Strains and plasmids are available upon request. Table S2 and Table S3 list the genotypes of all strains and plasmids, respectively, used in this study. File S1, File S2, File S3, and File S4 contain all the quantitative data from the screens described in this study.

## Results

### A proteome-wide assay for Mad2-dependent arrest

**Figure 1:**
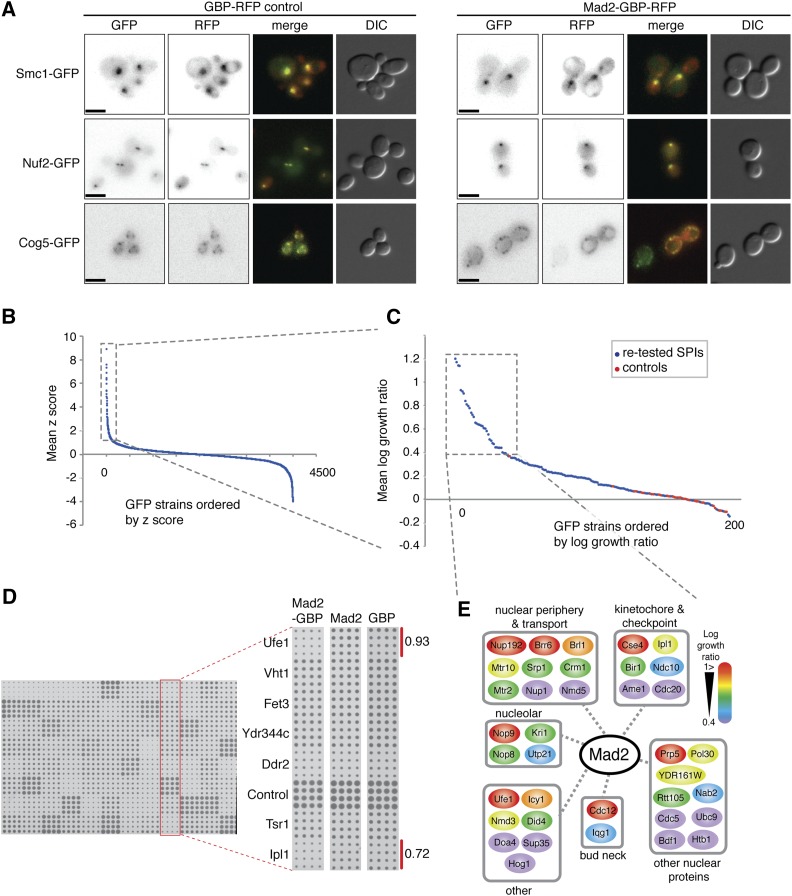
Forced relocalization of Mad2 in live cells. (A) Plasmids encoding either the GFP binding protein (GBP) or Mad2-GBP were transformed into yeast that express Smc1-GFP, Nuf2-GFP, and Cog5-GFP. In all cases, the GBP or Mad2-GBP colocalizes with the GFP. The scale bar is 5 µm. (B) The average effect upon growth of Mad2-GBP, compared with both Mad2 alone and GBP alone, is plotted for each GFP strain. The positive z score (standard score) indicates a growth defect. Experiments were performed with four replicates, and a z score of 1 indicates ∼1 SD from the mean of the proteome-wide data. (File S1). (C) The 156 most growth-restricted GFP strains with Mad2-GBP were retested with 16 replicates to confirm the growth defects. Log growth ratio (LGRs) > 0.4 indicate that colonies with the Mad2-GBP are < two-thirds the size of the average of the two controls (File S1). Included in these data are control strains (BY4741) that do not contain a fluorescent tag. (D) An example of the retested strains is shown; the highlighted column shows two GFP strains (*IPL1-GFP* and *UFE1-GFP*) that are growth restricted relative to controls—their LGRs are shown. (E) A schematic shows the 37 GFP strains that were growth inhibited by Mad2-GBP, grouped according to function, and color-coded according to the growth defect caused by their interaction (red interactions are strongest).

Activation of the SAC is thought to occur primarily at the kinetochore; however, there is evidence to support kinetochore-independent SAC activation (Fraschini *et al.* 2001; Poddar *et al.* 2005; Kim and Burke 2008; Rodriguez-Bravo *et al.* 2014), and roles for SAC proteins in other cellular compartments (Iouk *et al.* 2002; Wan *et al.* 2014). Additionally, there is a precedent for checkpoint activation via kinetochore-independent clustering of the Aurora kinase (Campbell and Desai 2013). Therefore, we hypothesized that associations of a checkpoint protein with a variety of cellular proteins could potentially activate the SAC. However, more specifically, we expected kinetochore associations to be especially effective for SAC activation. To systematically identify such associations we made use of SPI technology to create binary links between Mad2 and most other members of the proteome (Ólafsson and Thorpe 2015). In brief, ectopically expressed *MAD2* is linked to the sequence encoding a GFP-binding protein (GBP, (Rothbauer *et al.* 2006, 2008)), which also includes a red fluorescent protein (RFP) tag. The plasmid-encoded Mad2-GBP rescues the benomyl sensitivity of a *mad2∆* strain, indicating that it encodes a functional protein (Figure S1A). When transferred into GFP strains, the Mad2-GBP protein typically colocalizes with each GFP protein, consistent with strong binding between GBP and GFP (Figure 1A). Plasmids expressing *MAD2-GBP* or controls were transferred into the GFP collection of strains, with each strain assayed in quadruplicate, using SPA (Reid *et al.* 2011). The resulting plates were scanned and quantitatively assessed for growth using the ScreenMill suite of software to objectively quantify and compare colony sizes of experiments and controls (Dittmar *et al.* 2010). The rationale for this readout is that SAC activation will block growth. The two controls (*MAD2* alone or *GBP* alone) gave equivalent results (Figure S1B and File S1), hence we used an average growth score of Mad2-GBP relative to the two controls. We retested the strongest 156 interactions using the SPI methodology with 16 replicates per strain (Figure 1, C and D). This high-density retesting confirmed the strongest interactions (Figure S1C), and identified 37 GFP-tagged proteins that consistently produce a strong SPI phenotype with Mad2-GBP (Figure 1C, dashed box). A schematic of these Mad2 SPIs is shown in Figure 1E.

### SPIs are enriched for proteins at the nuclear periphery

Many of the Mad2 SPIs interact with each other, and include members of the network of genetic and physical interactions with Mad2 (Figure S1D). For example, two of the Mad2 SPIs, Cdc20, and Nab2, are known to have direct physical or genetic interactions with Mad2 (Batisse *et al.* 2009; Costanzo *et al.* 2010; Barford 2011). Also, three of the SPIs, Ame1, Cdc5, and Doa4, are encoded by genes with known genetic interactions with *MAD2* (Li *et al.* 1997; Daniel *et al.* 2006; Chiroli *et al.* 2009) (Figure S1D). The Mad2 SPIs are enriched for proteins whose gene ontology terms are enriched for function in nuclear transport and the kinetochore (*p*-values 2 × 10^−9^ and 1 × 10^−5^, respectively, Figure S1E) (Boyle *et al.* 2004). Mutants of the genes that encode the Mad2 SPIs are enriched for those affecting chromosome instability (Stirling *et al.* 2011) [Rank score/*p*-value = 5 × 10^−8^ (Thorpe *et al.* 2012)]. Thus, this Mad2 SPI screen has identified proteins that are associated with kinetochore function and chromosome segregation as well as proteins that are located at the nuclear periphery and involved in nuclear transport. There is evidence to show that checkpoint proteins play some role at the nuclear periphery, and that checkpoint activation may be dependent upon proteins normally at the nuclear periphery (Scott *et al.* 2009; Hou *et al.* 2012; Cairo *et al.* 2013). Additionally, one of the Mad2 SPIs is a nuclear protein encoded by an uncharacterized ORF, *YDR161W*.

In addition to the Mad2 interactions that block growth, we have identified a number of Mad2 interactions in our proteome-wide screen that increase growth relative to controls (the lower right tail in Figure 1B, and see File S1). We have not retested or investigated these interactions further, but we do note that Mad2 interaction with Smc3 (cohesin) and Stu1 (a microtubule binding protein) are among those interactions that enhance growth.

### Which Mad2-kinetochore associations block cell growth?

**Figure 2:**
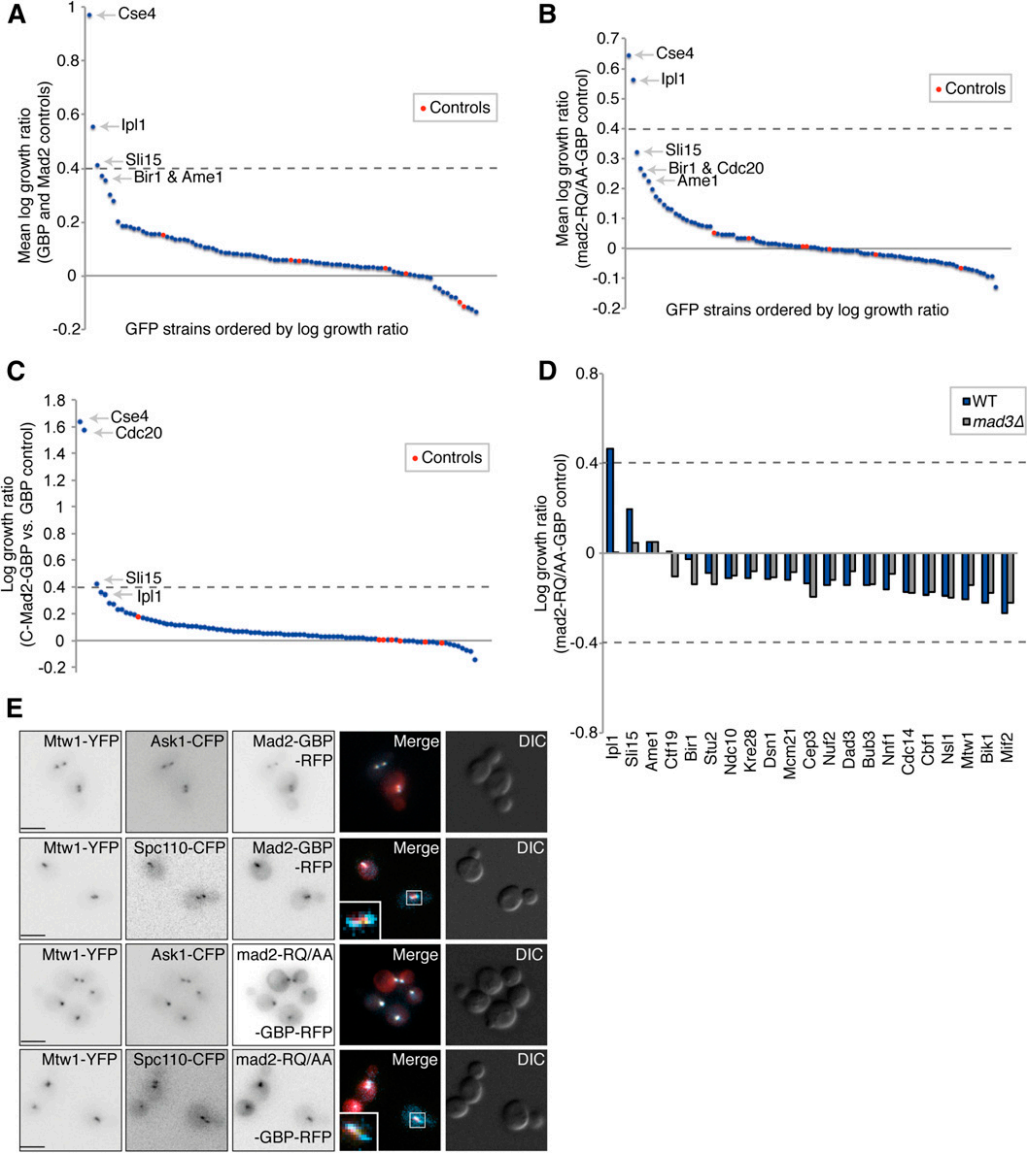
Cse4 is the only kinetochore protein to consistently cause a growth defect with Mad2 association. (A) A set of 88 kinetochore and kinetochore-associated GFP-tagged proteins was retested to confirm that Mad2 association does not lead to a growth defect. Mad2-GBP is compared with Mad2 alone and GBP alone. The dashed line indicates a mean LGR of 0.4 and the control strains are untagged BY4741, File S2. (B) The same set of 88 GFP strains were retested both with wild-type Mad2-GBP and mad2-RQ/AA-GBP, File S2. (C) The same set of 88 GFP strains were retested with closed Mad2-GBP (C-Mad2-GBP) and compared with GBP alone, File S2. (D) The *MAD3* gene was deleted from 21 of the GFP strains listed in (A) and (B). Wild-type *MAD3* and *mad3∆* GFP strains were retested for their sensitivity to Mad2-GBP and the mad2-RQ/AA-GBP control. The growth defects of Ipl1-GBP and Sli15-GFP with Mad2-GBP are suppressed by *mad3∆*. (E) Mad2-GBP and mad2-RQ/AA-GBP were imaged in cells encoding either Mtw1-YFP and Ask1-GFP, or Mtw1-YFP and Spc110-CFP. In all cases Mad2 colocalizes with Mtw1 (note that GBP does bind to YFP but not to CFP); scale bars are 5 µm.

Kinetochore recruitment of checkpoint proteins is often sufficient for SAC activation, even in the absence of unattached kinetochores (Jelluma *et al.* 2010; Maldonado and Kapoor 2011; Ito *et al.* 2012; Ballister *et al.* 2014; Aravamudhan *et al.* 2015). However, one striking observation from the SPI data is that most Mad2 associations to kinetochore proteins do not produce any changes in growth (File S1). We had expected a range of proteins at the outer kinetochore to be sufficient to activate the SAC via recruitment of Mad2. To confirm the proteome-wide data, we used a subset of 88 GFP strains, each of which encodes a different GFP-tagged kinetochore or kinetochore-associated protein (as listed in Table S1). These GFP strains were retested with Mad2-GBP in high-density format (16 replicates). This analysis reinforced the results of the proteome-wide analysis; the only kinetochore protein to produce a clear SPI phenotype when associated with Mad2 is Cse4 (Figure 2A and File S2). In addition, Ipl1 and, to a lesser extent, Sli15—two members of the chromosomal passenger complex (CPC)—also gave a weak SPI phenotype with Mad2. Ame1 and Ndc10, which we had identified as weak interactions in our proteome-wide screen, were below our threshold in this kinetochore-specific screen (log growth ratio < 0.4, experimental colonies are more than two-thirds the size of control colonies).

### Mad2 mutant controls

To try to detect more subtle effects and to control for indirect checkpoint activation we employed mutants of *MAD2* as controls for the kinetochore SPI screen. We created a mutant of *MAD2* (*mad2-R126,Q127/AA*) that encodes a dimerization mutant that binds normally to Mad1 but does not form an active MCC as it fails to convert O-Mad2 to a form that can bind Cdc20 (Nezi *et al.* 2006). We also created a *mad2∆C* mutant lacking the C-terminal 10 amino acids; this mutant is unable to form part of the MCC or bind Mad1 (Nezi *et al.* 2006). We then fused these mutant *mad2* genes to GBP to make constructs encoding Mad2-RQ/AA-GBP and Mad2∆C-GBP. We rescreened the kinetochore GFP strains comparing wild-type *MAD2-GBP* with these *MAD2-GBP* mutants. With either control, the set of SPIs identified remains essentially the same as with the standard controls (Figure 2B and Figure S2A). There are no growth defects with outer kinetochore components, the growth defect with Cse4 remains (Figure S2G), and only weak growth defects are seen with CPC members. In addition, the effect of Cdc20 linked to wild-type Mad2-GBP is striking when compared with Cdc20 linked to the mad2∆C-GBP control, consistent with the mad2∆C protein’s inability to contribute to the MCC (Figure S2A). It has been reported that forcing an active form of Mad2 (closed Mad2 or C-Mad2) to the kinetochore leads to metaphase arrest in human cells (Kruse *et al.* 2014). We therefore wanted to investigate if C-Mad2-GBP [mad2-L7A-GBP (Mapelli *et al.* 2007; Yang *et al.* 2008)] would be able to activate the SAC in cells with GFP-tagged kinetochore proteins. However, as seen with Mad2-GBP, the C-Mad2-GBP produced a SPI phenotype only with Cse4 and Cdc20, and, to a lesser extent, with Sli15 and Ipl1 (Figure 2C). In addition, we tested an additional *MAD2* mutant; mad2-T133A,V188N-GBP—a constitutively open form of Mad2 that does not bind open or closed forms of Mad2 (Mapelli *et al.* 2007)—and this failed to activate the SAC, even in association with Cdc20 or Ipl1 (Figure S2I). We find that the wild-type, closed form of Mad2 (C-Mad2) and dimerization-mutant Mad2 (mad2-RQ) arrest cells when associated with Cdc20, but the open form (mad2-TA,VN) and mad2∆C do not (Figure S2I).

To test whether any of the interactions were functioning through the checkpoint, we tested whether these SPIs could be suppressed by deletion of the *MAD3* gene that encodes a downstream component of the MCC. We repeated the assay both in wild-type and *mad3∆* versions of 21 of the GFP strains from the kinetochore SPI screen, and found that both the Ipl1 and Sli15 SPIs with Mad2 are suppressed (Figure 2D and Figure S2, B and H). The Mad2-Ipl1 and Mad2-Bir1 SPIs do result from colocalization of the two proteins (Figure S2, C and D, respectively). The Cse4-Mad2 SPI is not included in these data as the strains grew poorly and hence we analyzed this interaction using a different approach (discussed below).

We have previously found that associating kinetochore proteins with other proteins can result in their relocation away from the kinetochore (Ólafsson and Thorpe 2015), hence providing a possible reason why the Mad2-kinetochore associations failed to activate the checkpoint. However, we confirmed that Mad2-GBP and the mutant controls are recruited to a variety of mid and outer kinetochore proteins (Figure 1A, Figure 2E, and Figure S2, E and F).

Cse4 was the only kinetochore protein, when associated with Mad2, that consistently led to a growth defect (Figure 2, A and B). However, Cse4 is an unlikely target for Mad2-dependent SAC activation. Cse4 (CENP-A) is a centromere-specific histone 3 ortholog that forms part of a centromere-specific nucleosome, and is required for kinetochore assembly (Stoler *et al.* 1995; Collins *et al.* 2005). In contrast, normal SAC activation occurs via Mad1-Mad2 interaction with Bub1/3 bound to Spc105 (KNL1) at the outer kinetochore. Therefore, we suspected that the association of Cse4 with Mad2 was leading to a kinetochore defect, which either directly caused the growth phenotype, or indirectly activates the checkpoint. To differentiate between these two possibilities we asked whether or not the arrest could be rescued by deleting checkpoint components. To achieve this, we directly fused *CSE4* with *MAD2* under the control of a conditional promoter (*GAL1p*). Both N- and C-terminal *CSE4* fusions were made (*MAD2-CSE4* and *CSE4-MAD2*, respectively). Expression of both of these fusions arrests growth (Figure S3A), and we find that deletion of *MAD1* or *MAD3* is sufficient to rescue the arrest of the *MAD2-CSE4* fusion but not the reverse fusion (Figure S3B). Consistent with checkpoint activation, expression of the *MAD2-CSE4* fusion causes stabilization of cohesin (Figure S3, C and D). Furthermore, we find that expression of the *MAD2-CSE4* fusion does not lead to increased chromosome or plasmid loss, whereas the reverse fusion (*CSE4-MAD2*) increased chromosome loss (Figure S3, E and F). Since *MAD1* is normally required for Mad2 recruitment, the *mad1∆* rescue of the growth defect of Mad2-Cse4 suggests that the fusion is disrupting kinetochore function, and this, in turn, elicits checkpoint activation. Fusions to Cse4 in yeast have been shown to perturb normal kinetochore function (Wisniewski *et al.* 2014), as have changes to *CENP-A* homologs in other species (Ravi and Chan 2010). An internal fusion of GFP to Cse4 has been generated, which appears to function normally (Chen *et al.* 2000; Wisniewski *et al.* 2014). We therefore repeated the SPI assay with internally tagged Cse4 (*CSE4-GFP^int^*), and found no growth inhibition with Mad2-GBP, despite efficient recruitment of Mad2 to Cse4 (Figure S3, G and H). We also find that the high levels of expression achieved with the *GAL1* promoter produce a strong growth defect with Cse4-GFP bound to GBP alone, consistent with the notion that protein fusions with Cse4 can lead to growth defects (Figure S3G). Collectively, these data support the notion that the growth defect caused by linking Mad2 to the C-terminus of Cse4 (Cse4-Mad2) is a result of disrupting the normal structure and/or function of the kinetochore, which leads to chromosome instability and is independent of checkpoint activation. In contrast, linking Mad2 to the N-terminus of Cse4 (Mad2-Cse4) results in artificial checkpoint activation. However, this phenotype cannot be recapitulated with an internally tagged Cse4, or by recruiting Mad2 locally to other proteins at the inner kinetochore, suggesting that the checkpoint activation of Mad2-Cse4 is not specifically caused by the recruitment of Mad2 to the centromeric nucleosome. Thus, having excluded Cse4, none of the Mad2 associations with kinetochore proteins produced a significant growth defect using the SPI methodology.

### Mad1 recruitment to the kinetochore

Since the SPI data for Mad2 failed to identify outer kinetochore components, we decided to create kinetochore associations with the checkpoint component Mad1, which functions upstream of Mad2. Mad1 binds to phosphorylated Bub1 and then recruits Mad2 to kinetochores (London and Biggins 2014). As controls we used GBP alone, Mad1 alone and three different mutants of *MAD1*. The *mad1-RLK653-655/AAA* mutant encodes a protein that lacks the ability to bind Bub1 (Brady and Hardwick 2000), and is also incapable of activating the checkpoint (Ballister *et al.* 2014; Heinrich *et al.* 2014). The *mad1-RIL581-583/AAA* mutant encodes a protein that fails to bind Mad2 (Luo *et al.* 2002), and consequently should not activate a checkpoint response. Finally, *mad1-A736T* (*mad1-2*) acts as a *mad1∆* mutant likely via its failure to bind or activate Mad2 (Chen *et al.* 1999). The fusion of Mad1 to GBP does not affect its function as judged by the ability of *MAD1-GBP* to complement the benomyl sensitivity of a *mad1∆* strain (Figure S4A).

**Figure 3:**
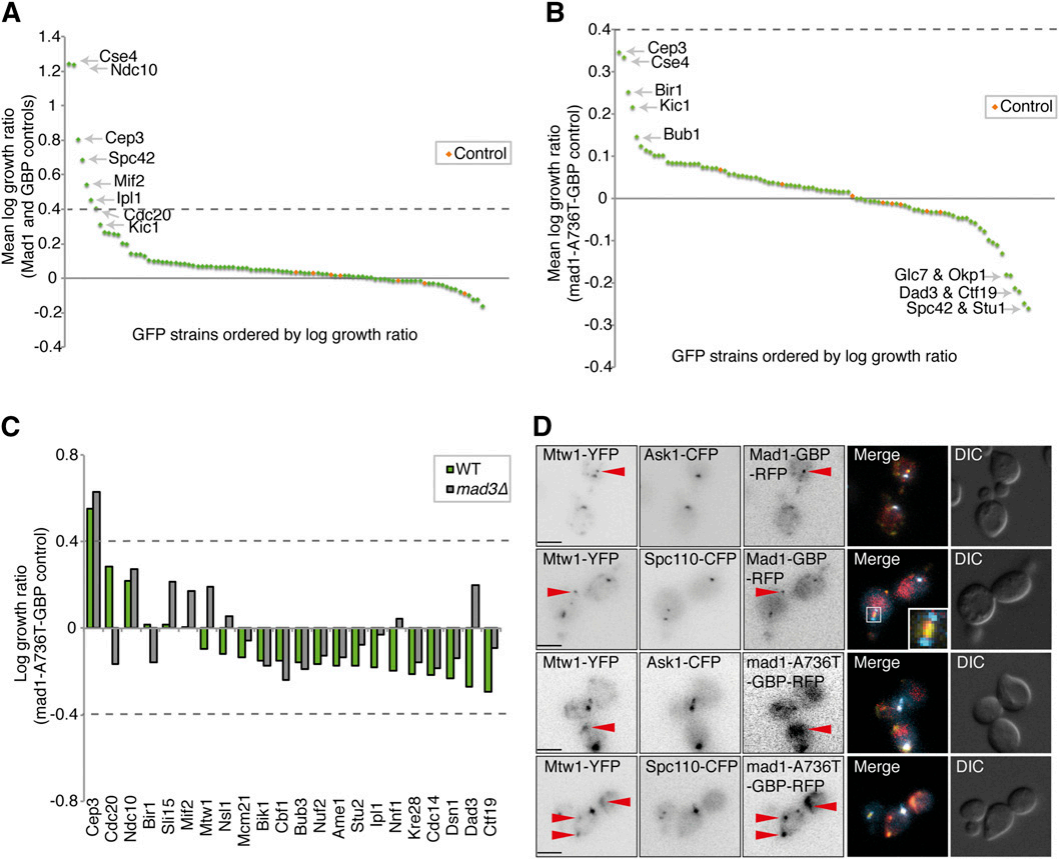
Growth effects of Mad1-kinetochore associations. (A) The 88 GFP-tagged kinetochore-related strains were tested for the effect of Mad1-GBP compared to the average of Mad1 alone and GBP alone. The experiment was performed with 16 replicates, and a number of GFP strains show detectable growth defects. The control strains, indicated in red, were untagged BY4741 (File S3). (B) The growth effects of Mad1-GBP are minimal when compared with an inactive mutant, mad1-A736T-GBP. (C) Mad1-GBP and mad1-A736T-GBP were transferred into 22 GFP strains both with and without the *MAD3* gene. The only significant Mad1 SPI, with Cep3, is not suppressed in *mad3∆* cells. (D) Fluorescence images of Mad1-GBP or mad1-A736T-GBP in Mtw1-YFP strains that also included either Ask1-CFP or Spc110-CFP (note that GBP does bind to YFP but not to CFP). In many cells there is a distinct population of Mad1-GBP and Mtw1-YFP that localizes distal to the kinetochore (red arrowheads); scale bars are 5 µm.

We tested the ability of Mad1-GBP to arrest cells encoding kinetochore proteins tagged with GFP using the methodology described above. We screened the 88 GFP strains that include kinetochore and kinetochore-associated proteins (Table S1). Mad1 associations with Cse4, Ndc10, Cep3, Spc42, Mif2, and Ipl1 all caused a growth defect (Figure 3A and File S3) with standard controls. However, using the mad1-A736T mutant protein as control, none of these interactions significantly affected growth (log growth ratio < 0.4, Figure 3B). Also, when comparing wild-type Mad1-GBP with either the mad1-RLK/AAA-GBP or the mad1-RIL/AAA-GBP controls, there were no significant growth defects (Figure S4, B and C, respectively). To assess whether any of the subtle growth defects were related to checkpoint activation, we repeated the SPI assay in both wild-type and *mad3∆* versions of 22 of the GFP strains. The only weak SPI to be suppressed by *mad3∆* was between Mad1 and Cdc20 (Figure 3C). The Mad1-Cep3 interaction (Figure S4D) was not suppressed by *mad3∆* (Figure 3C), indicating that the effect of this interaction is independent of checkpoint activation. To confirm that the Mad1-GBP is recruited to kinetochores via the GBP–GFP interaction, we imaged a number of GFP-kinetochore strains with Mad1-GBP. We found that Mad1-GBP localizes to kinetochore-bound GFP proteins (Figure 3D and Figure S5, A and B), although in many cells, a fraction of the Mad1 and GFP-tagged kinetochore protein is displaced from the endogenous kinetochore (Figure 3D, red arrows); however, we note that this does not cause a detectable growth defect.

**Figure 4:**
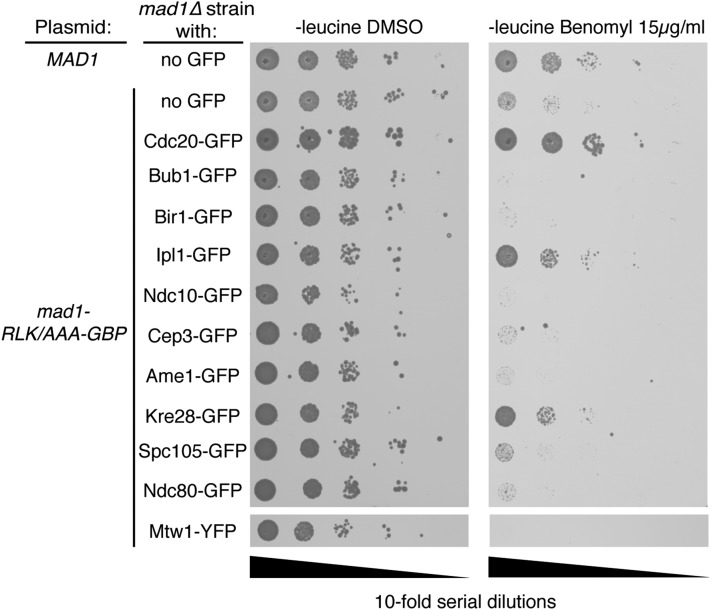
The mad1-RLK-AAA mutant participates in a SAC response when associated with Kre28. Serial dilutions of *mad1∆* GFP strains shows that cells with a plasmid encoding mutant mad1-RLK/AAA-GBP, which normally does not complement *mad1∆*, rescues benomyl sensitivity in cells containing GFP-tagged Kre28, Cdc20, and Ipl1.

Together with the Mad2 data, these Mad1 data are consistent with the notion that locally high concentrations of either Mad1 or Mad2 at the kinetochore alone are not sufficient for checkpoint activation. Another possibility is that the GFP–GBP interaction prevents checkpoint proteins from being in the precise location or orientation for checkpoint activation. We tested this notion by examining the effect of linker length. We typically use an eight amino acid flexible linker between the N-terminus of GBP and the C-terminus of our target protein (Mad2 or Mad1). To test if this linker was potentially too long, we created a set of *MAD1-GBP* constructs (wild type and mutants) that have a four amino acid linker to restrict the movement of the Mad1 protein more closely to the GFP tagged protein (Mad1-4A-GBP). However, there were minimal differences on the SPI results upon changing linker length (Figure S4, E–H). Although Mad1-GBP rescued the benomyl sensitivity of a *mad1∆* strain (Figure S4A), this protein was not tethered to a GFP-tagged protein. To further test whether the GFP-GBP interaction is preventing checkpoint activation, we tested whether a Mad1 tethered to the kinetochore via GBP was capable of participating in checkpoint activation. We have shown that a construct encoding mad1-RLK/AAA-GBP is unable to rescue the checkpoint response in a *mad1∆* strain (Figure S4A), likely due to its inability to bind Bub1, and thus be recruited to kinetochores (Brady and Hardwick 2000). However, we reasoned that if we used the GBP–GFP system to recruit mad1-RLK/AAA adjacent to Spc105 (KNL1), it might participate in a normal checkpoint response. We tested this notion by asking whether mad1-RLK/AAA-GBP tethered to GFP-tagged kinetochore proteins in close proximity to Spc105 could rescue the benomyl sensitivity of *mad1∆* strains. We find that *mad1∆* strains are resistant to benomyl if mad1-RLK/AAA is recruited to a subunit of the SPC105 complex, Kre28, but not several other kinetochore proteins, including Spc105 itself (Figure 4). In addition, *mad1∆* benomyl sensitivity is also rescued by associating mad1-RLK/AAA with Cdc20 and Ipl1. These data show that the GBP-tethered Mad1 is capable of participating in a checkpoint response.

### Mps1 kinetochore recruitment

**Figure 5:**
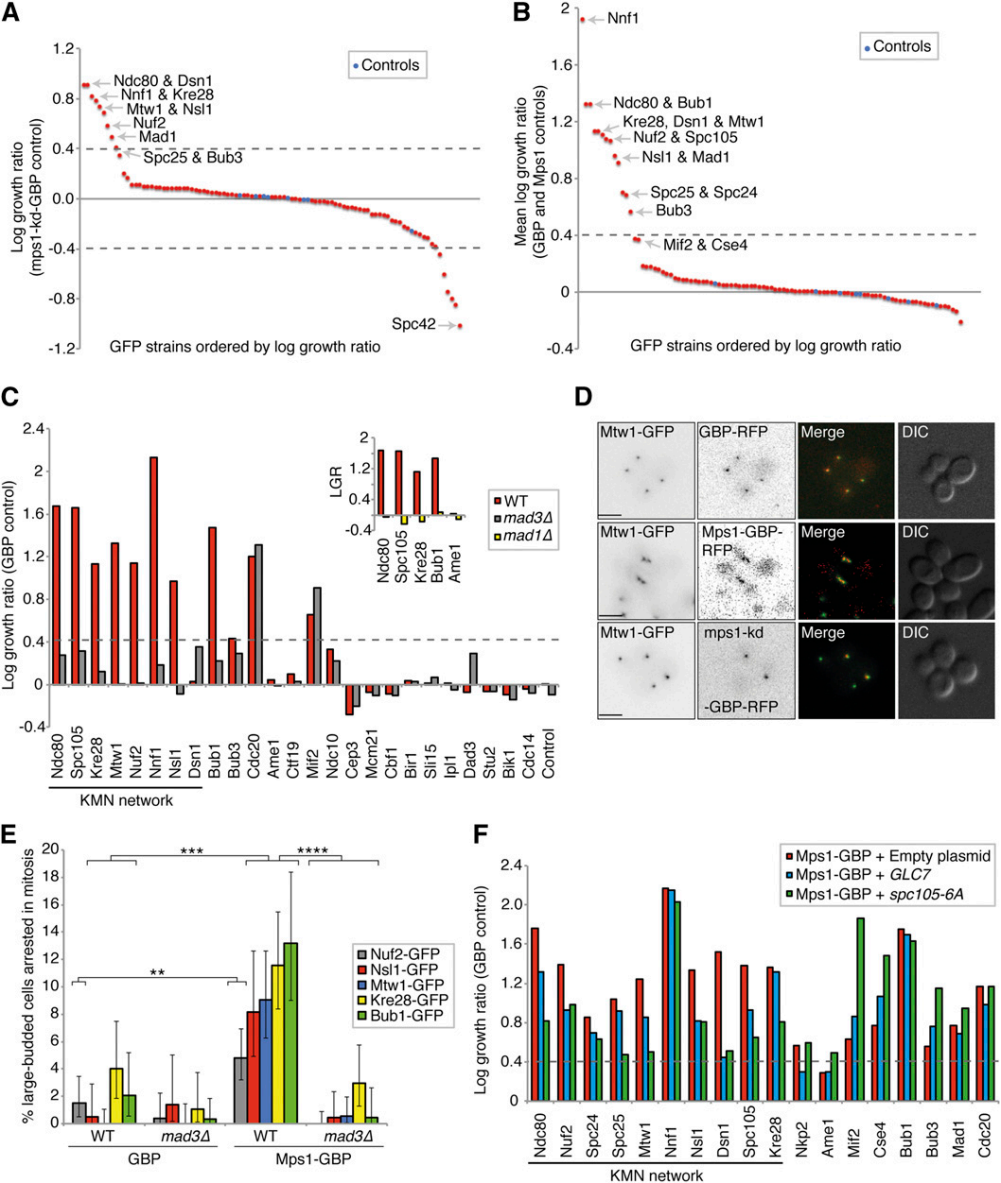
Mps1 recruitment to the KMN network results in a growth defect. (A) Mps1-GBP and a kinase-dead control (mps1-kd-GBP) were transferred into the 88 kinetochore-related GFP strains. A number of GFP strains show growth defects, including most members of the KMN network of proteins. The strains labeled ‘Controls’ in blue are an untagged BY4741 strain (File S4). (B) We also compared Mps1-GBP with two other controls (Mps1 or GBP alone), and all of the KMN network of proteins produce a growth defect (File S4). (C) We deleted the *MAD3* gene in 25 of these GFP strains and repeated the assay with Mps1-GBP compared with GBP alone. (Inset) We also deleted *MAD1* in several GFP strains. The KMN-Mps1 (and Bub1) SPIs are all suppressed by deletion of *MAD3* and *MAD1* (except Dsn1). LGR, log growth ratio. (D) Fluorescence image analysis confirms the colocalization of Mps1 with the KMN protein Mtw1; scale bars are 5 µm. (E) Cell cycle progression analysis of Mps1 SPIs. Colonies from the SPI assay were grown in culture overnight, imaged the following day with fluorescent microscopy, and cells counted (ranging from 141 to 560 cells for each condition). The graph shows the percentage of large-budded cells with two GFP-kinetochore foci in close proximity. Large-budded cells with separated kinetochore foci were discounted. Statistical analysis was done using Fishers exact test; **** *p* < 0.0001. *** *p* < 0.001, ** *p* < 0.01. Error bars indicate 95% binomial C.I. (F) Mps1-GBP and the GBP control were transferred into the 88 kinetochore-related GFP strains. In addition, three separate plasmids were also transferred simultaneously into these GFP strains, one empty, one with *pCUP1-GLC7*, and the third with *pGALS-spc105-6A*. The graph shows a selection of the resulting data, and shows that both increased expression of *GLC7* (blue bars), and overexpression of *spc105-6A* (green bars), suppress the Mps1 SPI growth phenotype specifically at the KMN network compared to the empty plasmid (red bars).

**Figure 6:**
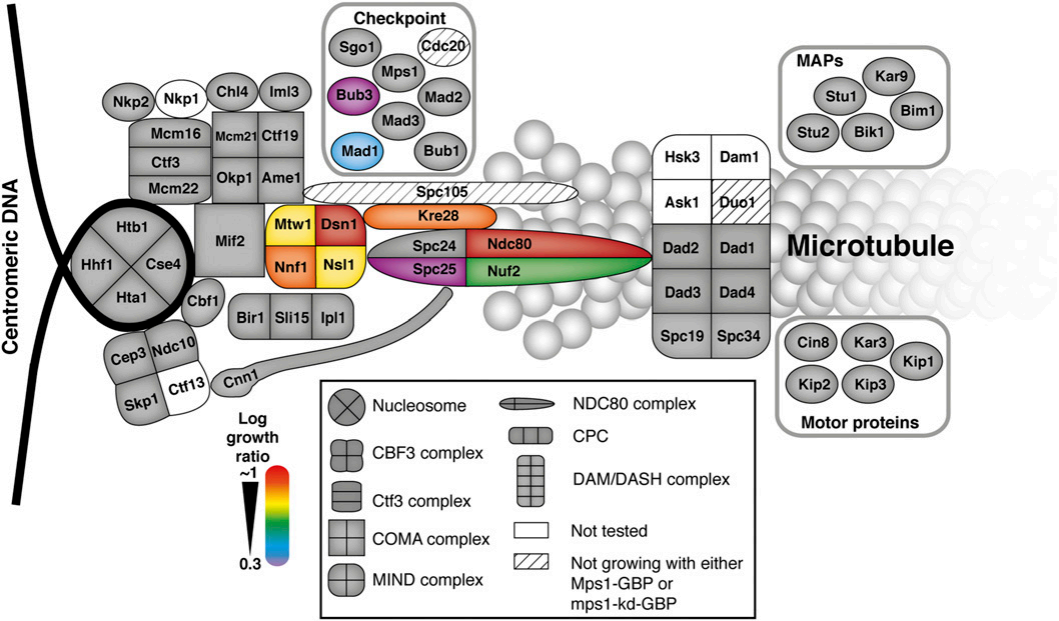
Map of Mps1 kinetochore SPIs. A schematic of the kinetochore proteins illustrates those proteins that when associated with active Mps1 lead to growth arrest. These proteins are predominantly within the KMN network of mid to outer kinetochore proteins, adjacent to Spc105. The strength of the SPI is color-coded, red strongest, purple weakest.

Since neither Mad1 nor Mad2 were able to activate the checkpoint when recruited to kinetochore proteins, we asked whether recruitment of the checkpoint kinase Mps1 would be sufficient to drive checkpoint activation. We fused *MPS1* to GBP and repeated the kinetochore SPI screen with this construct. As a control we used a kinase dead mutant *mps1-D580A-GBP* (*mps1-kd*) (Lauze *et al.* 1995; Araki *et al.* 2010) in addition to the Mps1 and GBP controls. Fusion of Mps1 to GBP did not abrogate its function, as judged by our ability to replace the endogenous *MPS1* allele, an essential gene, with the *MPS1-GBP* allele. In contrast to Mad1 and Mad2, recruitment of Mps1 is sufficient to block cell growth with most members of the KMN network including Ndc80, Dsn1, Nnf1, Kre28, Mtw1, Nsl1, Nuf2, and also Mad1 (Figure 5A, Figure 6, and File S4). When comparing experiments against the GBP and Mps1 controls, all members of the KMN network and Bub1, Bub3, and Mad1, affect growth when associated with Mps1 (Figure 5B). To confirm that these interactions are causing checkpoint activation, we retested some of these SPIs in *mad3∆* cells (including Ndc80, Nuf2, Spc105, Kre28, and the MIND complex members). Deletion of the *MAD3* gene rescued the growth arrest of the Mps1-KMN network interactions, and *mad1∆* also rescued the SPI phenotype of Ndc80, Spc105, Kre28, and Bub1, consistent with these Mps1 SPIs resulting from checkpoint activation (Figure 5C and Figure S5, C and D). To confirm that Mps1-GBP is recruited to a number of different GFP-tagged kinetochore proteins, we examined cells using fluorescence imaging. We find that the Mps1-GBP is colocalized with the GFP protein (Figure 5D and Figure S5, E–G). In addition, we measured the cell cycle progression of five different Mps1 SPIs: Nuf2, Nsl1, Mtw1, Kre28, and Bub1. We quantified large-budded cells that display two GFP foci in close proximity, representing cells in mitotic arrest, and show that all five GFP strains measured have an increased frequency of large-budded cells when expressing *MPS1-GBP* vs. *GBP* alone (Figure 5E). Confirming that this mitotic arrest is caused by SAC activation, we found that the large-budded cell phenotype is completely rescued in *mad3∆* cells.

Since Mps1 kinase activity seems to be the key driver of SAC activation, we asked if we could suppress the SPI phenotype caused by the constitutive Mps1 kinetochore recruitment by increasing the expression of the antagonizing phosphatase, *GLC7*, which is required for SAC silencing (Rosenberg *et al.* 2011). We repeated the Mps1 kinetochore SPI assay, but now included a second separate plasmid expressing *GLC7* under the control of *pCUP1*. This plasmid was introduced into cells along with the Mps1-GBP plasmid. The same *GLC7* plasmid was also introduced into the control strains with the GBP plasmid to control for the ectopic expression of *GLC7*. Increasing Glc7 in cells did suppress the growth defect of some Mps1-kinetochore SPIs, namely Ndc80, Nuf2, Mtw1, Nsl1, Dsn1, and Spc105, which are all members of the KMN network as seen by comparing with cells containing Mps1-GBP plus an empty plasmid (Figure 5F). Furthermore, we reasoned if Mps1 activates the SAC at the kinetochore through phosphorylation of MELT repeats on Spc105 (which Glc7 dephosphorylates) we should also see suppression or rescue of the SPI growth phenotype by expressing a mutant of *SPC105* (*spc105-6A*) that has the MELT repeats mutated so that they cannot be phosphorylated (London *et al.* 2012; Primorac *et al.* 2013). As before, we introduced a separate plasmid into cells with the Mps1-GBP plasmid, now expressing *spc105-6A* mutant under the control of *pGALS* to overexpress it in order to compete against the endogenous Spc105. To control for the overexpression of *spc105-6A*, this plasmid was also introduced into the GBP control cells. Compared to GFP strains containing Mps1-GBP and an empty plasmid, all the KMN-GFP strains containing both Mps1-GBP and spc105-6A had a reduced growth defect (Figure 5F). Interestingly, neither Bub1 or Bub3 association with Mps1 is suppressed by either of these plasmids (*spc105-6A* or *GLC7*), despite being rescued by *mad3∆*. This suggests that activation of the SAC via forced association of Mps1 with Bub1 (an Mps1 target) can act independently from the Spc105 phosphorylated MELT motifs.

In summary, compared with a kinase-dead Mps1, an active kinase arrests cell division when recruited to most members of the KMN network (Figure 6), and the arrest is consistent with checkpoint activation.

## Discussion

In previous studies to identify which proteins, when associated with the yeast kinetochore (Mtw1, Dad2, and Nuf2), would cause a growth arrest (Ólafsson and Thorpe 2015; Berry *et al.* 2016), we were surprised not to identify any of the key checkpoint proteins (Bub1, Bub3, Mad1, Mad2, and Mad3 were included in these screens). The activation of the SAC at the kinetochore requires the recruitment of a set of checkpoint proteins, including the Mps1 kinase, Bub1, Bub3, Mad1, and Mad2. Therefore, we set out to test systematically whether checkpoint protein localization would be sufficient to activate the SAC. In a proteome-wide screen of Mad2 interactions, we identify interactions that cause a growth defect (SPIs) that are enriched for proteins related to kinetochore function and chromosome segregation, and also those located at the nuclear periphery. However, we failed to identify any kinetochore proteins except for Cse4. We think that the Cse4-Mad2 interaction is not direct activation of the checkpoint since C-terminal fusions to Cse4 are hypomorphic, and, when associated via GBP to Mad2, likely disrupts the structure of the inner kinetochore. Consistently, C-terminal fusions to Cse4 activate the checkpoint at high temperature (Ho *et al.* 2014). Our Cse4 data raise an interesting problem for making SAC-kinetochore associations. If a specific association leads to kinetochore defects, these in turn may activate the SAC. The defects may be partially relieved by deleting the SAC, but nevertheless do not represent normal SAC activation. This problem is difficult to resolve even for seemingly obvious cases, for example, fusing Cdc20 to Mad2 leads to arrest (Lau and Murray 2012; and Figure S2A), and these two proteins bind together as part of the MCC (Barford 2011); however, by fusing them, we cannot be sure whether we are creating a ‘normally’ active MCC or simply disrupting the function of Cdc20. The same argument could be used for other studies that induce SAC activation via kinetochore or checkpoint protein fusions or associations (Maldonado and Kapoor 2011; Kruse *et al.* 2014; Aravamudhan *et al.* 2015). To attempt to address this issue, we used mutants of *MAD2* in an effort to control for specific effects of the active version of the protein. However, we find that Cse4 remained a SPI even when comparing wild-type Mad2 with either mad2-RQ/AA-GBP or mad2∆C-GBP (Figure 2B and Figure S2A). We suggest that the wild-type Mad2 protein, unlike the mutant versions, is able to interact with other proteins, such as Mad1 and Cdc20, to form a large protein complex. These larger Mad2-aggregates then may be more effective at disrupting kinetochore structure when associated with Cse4 than an isolated Mad2 mutant protein.

We did not detect checkpoint activation when recruiting a constitutively active (closed) form of Mad2, which produces robust checkpoint activation in mammalian cells (Kruse *et al.* 2014). We cannot completely rule out that some aspect of the Mad2-GBP is refractory for checkpoint activation or there may be stoichiometric changes in Mad2 that prevent checkpoint activation (Heinrich *et al.* 2013); however, the Mad2-GBP does rescue benomyl sensitivity in a *mad2∆* strain. We find a similar failure to activate the SAC when Mad1 is recruited to kinetochores. Mad1-GBP rescues the benomyl sensitivity of a *mad1∆* strain, and changing the linker length between Mad1 and GBP did not facilitate checkpoint activation. More importantly, when we express *mad1-RLK/AAA-GBP* in *KRE28-GFP mad1∆* cells, it also rescues benomyl sensitivity; however, when *mad1-RLK/AAA-GBP* is expressed in non-GFP *mad1∆* cells, or expressed in *mad1∆* cells with GFP-tagged proteins elsewhere at the kinetochore (*e.g.* Ame1 or Ndc80), it does not rescue benomyl sensitivity (Figure 4). These data show that Mad1 tethered via the GBP-GFP interaction at the SPC105 complex can participate in checkpoint activation. We speculate that the binding of mad1-RLK/AAA-GBP to Bub1, Ndc80, or Spc105 fails to rescue benomyl sensitivity of *mad1∆* strains due to the requirement for accurate positioning of Mad1 within the kinetochore, which is not achieved with these associations (note that all GBP and GFP fusions are to the C-termini of these proteins, and the MELT repeats on Spc105 are at the N-terminus). It is possible that N-terminal fusions of GFP to kinetochore proteins might position the Mad1 or Mad2 in a better position for checkpoint activation; however, by testing so many C-terminal GFP fusions we are positioning Mad1 and Mad2 at most positions in the kinetochore. For example, the C-terminal domain of both the Spc24/25 dimer and Spc105 interacts with the MIND complex at the outer kinetochore. Conversely, the C-termini of both Dsn1 and Nnf1 face toward the inner kinetochore. Furthermore, the C-terminus of Mtw1 is close to the N-termini of Dsn1, Nnf1, and Nsl1 (Maskell *et al.* 2010). In addition, the C-terminus of the Spc24/25 dimer interacts with the N-terminus of Cnn1 (Malvezzi *et al.* 2013), and the MIND complex interacts with the N-termini of Ame1, Okp1, and Mif2 (Hornung *et al.* 2014).

The discrepancy between our data in *Saccharomyces cerevisiae* and those from other systems, notably the forced kinetochore association of Mad1 or Mad2, could point to an additional level of SAC control that is present in budding yeast but not in either fission yeast or human cells. Kinetochore recruitment of Mad1 and Mad2 can activate the SAC in mammalian cells (Maldonado and Kapoor 2011; Ballister *et al.* 2014; Kruse *et al.* 2014) and fission yeast (Heinrich *et al.* 2014), although recruitment of Bub1/3 is not sufficient for SAC activation without Mps1 activity (Rischitor *et al.* 2007; Ito *et al.* 2012; Yamagishi *et al.* 2012; London and Biggins 2014). In human cells, forced kinetochore recruitment of mad1-RLK/AAA still recruits Mad2, but does not activate the SAC, suggesting that Mad2 recruitment alone is not sufficient for artificial SAC activation (Ballister *et al.* 2014). Mps1 activity is also required to maintain the SAC response in human cells, even when Mad1 is artificially tethered to the kinetochore (Maldonado and Kapoor 2011). In addition, Mps1 catalytic activity enhances the production of closed Mad2 in human cells (Tipton *et al.* 2013), underlining the importance of Mps1 in maintaining a SAC response. Our data support a model that recruitment of Mad1 and Mad2, like Bub1/3, while necessary, is not sufficient for robust SAC activation. Previous work in *S. cerevisiae* has hinted at this finding; tethering the Mad1-binding domain of Bub1 to Spc105, which itself lacks Mps1 phosphorylation sites, increases recruitment of Mad1 to kinetochores but does not block cell division (London and Biggins 2014). Also, recruitment of Mad1 to Mtw1 did not block growth (Aravamudhan *et al.* 2015). Consistent with the SPI between Mps1 and Mad1, overexpression of *MPS1* results in hyperphosphorylation of Mad1 and SAC activation by forming a Mad1-Bub1-Bub3 complex (Hardwick *et al.* 1996; Brady and Hardwick 2000). It is possible that some of the Mad1 or Mad2 associations with the kinetochore do activate the checkpoint, but that the checkpoint is not maintained; our SPI system does not assay this, and this may be better assayed using a conditional interaction system. However, Mad1 recruitment to Mtw1 fails to activate the checkpoint using a conditional system (Aravamudhan *et al.* 2015). Our data are consistent with the kinase activity of Mps1 being the critical regulator of the SAC. However, one important implication of our experiments is that kinetochore–SAC associations can indirectly lead to SAC activation (as seen for Mad2-Cse4), and attempts to differentiate such artifacts from true checkpoint activation using mutants are not always successful.

In contrast to Mad1 and Mad2, the checkpoint kinase Mps1 activated the SAC when recruited to the KMN network of proteins (Figure 5 and Figure 6), consistent with previous reports (Jelluma *et al.* 2010; Ito *et al.* 2012; Aravamudhan *et al.* 2015; Dou *et al.* 2015). A recent study found that recruitment of Mps1 to specific members of the DAM1/DASH complex (Dad2, Dad4, Spc19, and Spc34) activated the SAC; however, we did not detect this in our SPI screen (Dad1, Dad2, Dad3, Dad4, Spc19, and Spc34 were tested in this screen) (Aravamudhan *et al.* 2015). We note that, in this previous study, the growth effect of recruiting Mps1 to the DAM1/DASH was considerably weaker than recruiting Mps1 to Mtw1; hence, it is likely that the DAM1/DASH phenotype is not detected in our SPI screen. Alternatively, this study used a conditional association system (using rapamycin to associate FKBP12 with the FRB domain of mTOR) to direct protein association. It is possible that our system, which uses constitutive protein association may give different results, since cells may adapt to a persistent checkpoint signal. Nevertheless, our study maps the locations within the kinetochore at which Mps1 recruitment can activate the SAC, and this is consistent with the expected phosphorylation of the MELT repeats in Spc105. We cannot prove that Mps1 is directly phosphorylating Spc105; it is possible that either Ndc80 (Kemmler *et al.* 2009) or Cnn1 (Thapa *et al.* 2015) are phosphorylated. Alternatively, the Mps1 SPIs may result from inappropriate phoshorylations that affect kinetochore function and activate the SAC indirectly. However, expression of either a mutant that encodes a nonphosphorylatable form of Spc105, or a gene encoding the phosphatase, Glc7, that reverses checkpoint activation, suppressed these Mps1 SPIs, implying a direct effect of Mps1.

## Supplementary Material

Supplemental Material
