# Supplementary material for: Synthetic Physical Interactions Map Kinetochore-Checkpoint Activation Regions
Source: G3 (Bethesda). 2016 Jun 8;6(8):2531–42. doi: 10.1534/g3.116.031930 (PMC4978906; doi:10.1534/g3.116.031930)
Supplement: Supplemental Material [file supp_6_8_2531__index.html]

Synthetic Physical Interactions Map Kinetochore-Checkpoint Activation Regions — Supplemental Material 

# Synthetic Physical Interactions Map Kinetochore-Checkpoint Activation Regions

## Supplemental Material for Olafsson and Thorpe, 2016

**Files in this Data Supplement:**

- Figure S1 - Proteome-wide Mad2 SPI screen analysis. (.pdf, 445 KB)
- Figure S2 - Mad2 kinetochore SPIs. (.pdf, 523 KB)
- Figure S3 - Analysis of *MAD2-CSE4* direct fusion and Mad2-Cse4 SPI. (.pdf, 576 KB)
- Figure S4 - Mad1 kinetochore SPIs. (.pdf, 470 KB)
- Figure S5 - Further Mad1 and Mps1 kinetochore SPI analysis. (.pdf, 522 KB)
- File S1 - Results of the proteome wide Mad2 SPI screen and the retests of the GFP strains with the strongest growth defects. (.xlsx, 333 KB)
- File S2 - Results of the Mad2 SPI screens with the 88 GFP strains that represent the kinetochore and kinetochore associated proteins. (.xlsx, 63 KB)
- File S3 - Results of the Mad1 SPI screens with the 88 GFP strains that represent the kinetochore and kinetochore associated proteins. (.xlsx, 68 KB)
- File S4 - Results of the Mps1 SPI screens with the 88 GFP strains that represent the kinetochore and kinetochore associated proteins. (.xlsx, 56 KB)
- Table S1 - List of GFP strains used in our kinetochore-specific SPI screens. (.xlsx, 53 KB)
- Table S2 - Yeast strains used in this study. (.xlsx, 45 KB)
- Table S3 - Plasmids used in this study. (.xlsx, 39 KB)
