# Supplementary material for: Synthetic Physical Interactions Map Kinetochore-Checkpoint Activation Regions
Source: G3 (Bethesda). 2016 Jun 8;6(8):2531–42. doi: 10.1534/g3.116.031930 (PMC4978906; doi:10.1534/g3.116.031930)
Supplement: Supplemental Material [file supp_g3.116.031930_FigureS1.pdf]

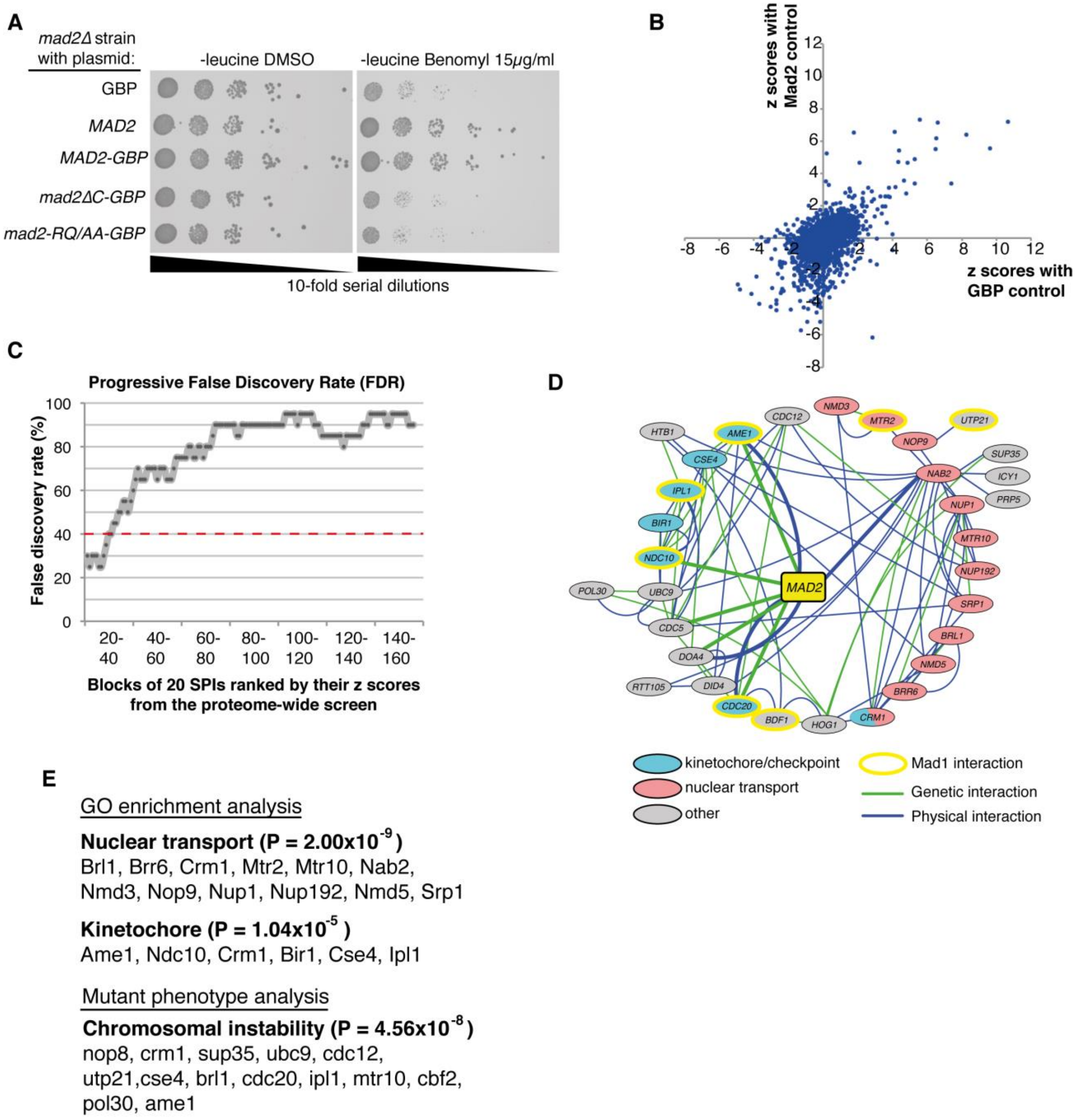

Figure S1 Proteome-wide Mad2 SPI screen analysis.

(A) Serial dilutions of *mad2Δ* strain shows that cells with plasmids containing *MAD2* or *MAD2-GBP* rescue benomyl sensitivity of *mad2Δ*, but not plasmids containing GBP, *mad2ΔC-GBP* or *mad2-RQ/AA-GBP*.

(B) The z scores from the two controls in the proteome-wide screen are plotted on each axis in a scatter graph (higher z scores represent a stronger growth defect relative to controls).

(C) The rate of false discovery increases as the strength of the SPI decreases.

(D) The Mad2 SPIs have extensive genetic and physical interactions (green and blue lines respectively). Color coding indicates the function of some of the SPIs; blue for kinetochore and spindle proteins and pink for proteins involved in nuclear transport. Mad2 SPIs that have either a genetic or physical interaction with Mad1 are highlighted in yellow.

(E) The 37 validated Mad2 SPIs are enriched for proteins that function in nuclear transport and at the kinetochore as assessed with gene ontology enrichment. Mutations in the genes encoding these Mad2 SPIs are enriched for those that produce a chromosomal instability phenotype.
