# Supplementary material for: Synthetic Physical Interactions Map Kinetochore-Checkpoint Activation Regions
Source: G3 (Bethesda). 2016 Jun 8;6(8):2531–42. doi: 10.1534/g3.116.031930 (PMC4978906; doi:10.1534/g3.116.031930)
Supplement: Supplemental Material [file supp_g3.116.031930_FigureS2.pdf]

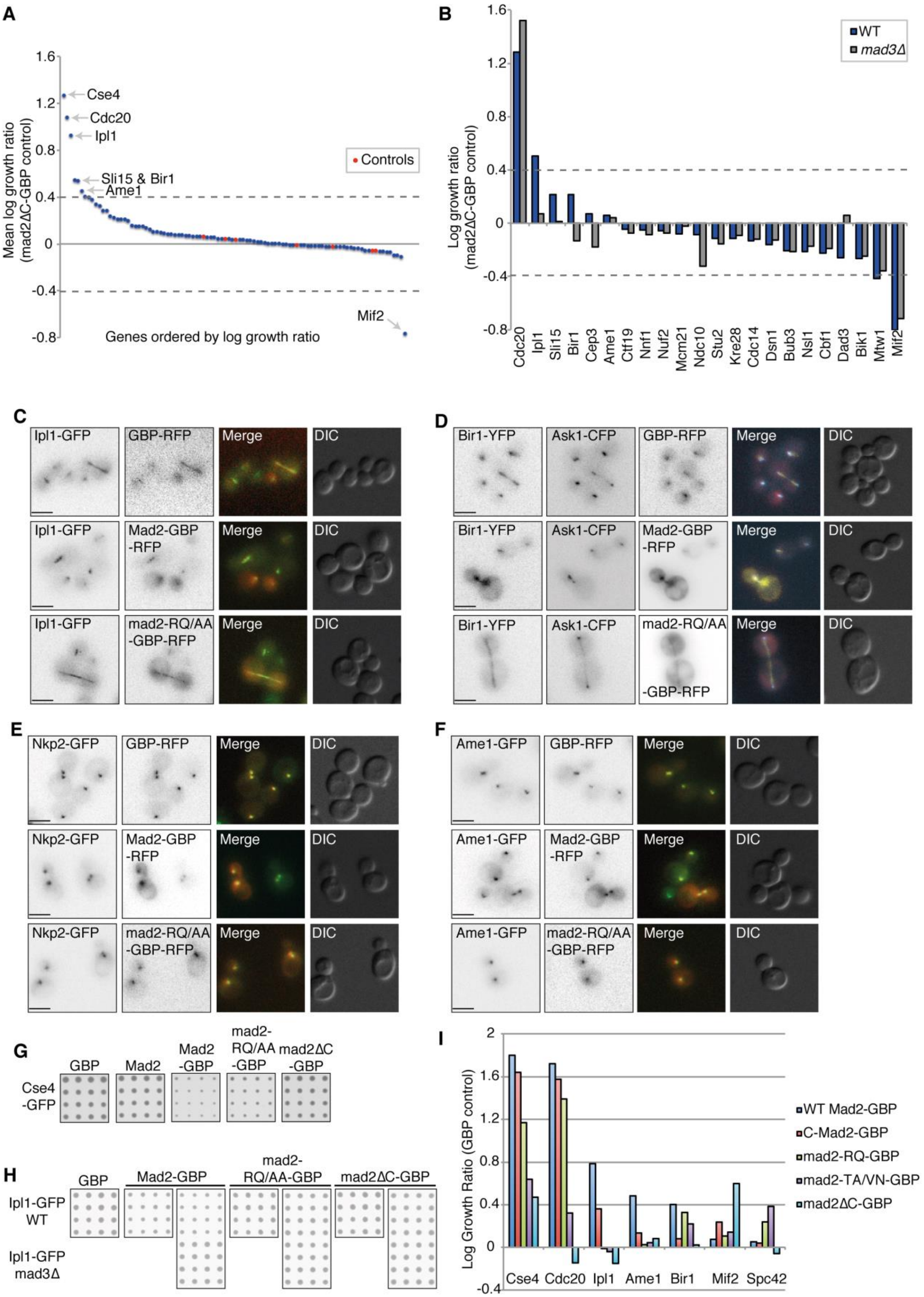

A
Mean log growth ratio (mad2ΔC-GBP control)
1.6
1.2
0.8
0.4
0
-0.4
-0.8
Cse4
Cdc20
Ipl1
Sli15 & Bir1
Ame1
Controls
Mif2
Genes ordered by log growth ratio
B
Log growth ratio (mad2ΔC-GBP control)
WT
mad3Δ
Cdc20
Ipl1
Sli15
Bir1
Cep3
Ame1
Ctf19
Nnf1
Nuf2
Mcm21
Ndc10
Stu2
Kre28
Cdc14
Dsn1
Bub3
Nsl1
Cbf1
Dad3
Bik1
Mtw1
Mif2
C
Ipl1-GFP
GBP-RFP
Merge
DIC
Mad2-GBP -RFP
mad2-RQ/AA -GBP-RFP
D
Bir1-YFP
Ask1-CFP
GBP-RFP
Merge
DIC
Mad2-GBP -RFP
mad2-RQ/AA -GBP-RFP
E
Nkp2-GFP
GBP-RFP
Merge
DIC
Mad2-GBP -RFP
mad2-RQ/AA -GBP-RFP
F
Ame1-GFP
GBP-RFP
Merge
DIC
Mad2-GBP -RFP
mad2-RQ/AA -GBP-RFP
G
GBP
Mad2
Mad2 -GBP
mad2- RQ/AA -GBP
mad2ΔC -GBP
Cse4 -GFP
H
GBP
Mad2-GBP
mad2- RQ/AA-GBP
mad2ΔC-GBP
Ipl1-GFP WT
Ipl1-GFP mad3Δ
I
Log Growth Ratio (GBP control)
2
1.6
1.2
0.8
0.4
0
-0.4
Cse4
Cdc20
Ipl1
Ame1
Bir1
Mif2
Spc42
WT Mad2-GBP
C-Mad2-GBP
mad2-RQ-GBP
mad2-TA/VN-GBP
mad2ΔC-GBP

Figure S2 Mad2 kinetochore SPIs.

(A) A set of 88 kinetochore and kinetochore-associated GFP-tagged proteins were retested (Table S1). Mad2-GBP is compared with mad2ΔC-GBP. The dashed line indicates a mean log growth ratio (LGR) of 0.4 and the control strains, indicated in red, are untagged BY4741, File S2.

(B) The *MAD3* gene was deleted from 22 GFP-tagged kinetochore strains and the WT and *mad3Δ* strains were retested for their sensitivity to Mad2-GBP versus the mad2ΔC-GBP control. The growth defects of Ipl1-GFP, Sli15-GFP and Bir1-GFP with Mad2-GBP are suppressed by *mad3Δ* (File S2).

(C-F) Imaging of cells containing Mad2-GBP, mad2-RQ/AA or GBP plasmids and GFP/YFP-tagged proteins shows that Mad2-GBP and mad2-RQ/AA-GBP are recruited to Ipl1-GFP (C), Bir1-YFP (D) (note that GBP does not bind CFP), Nkp2-GFP (E), and Ame1-GFP (F). Fluorescence contrast differs between images and the scale bar is 5 µm.

(G) The growth effects of the Cse4-Mad2 SPI (and controls) are shown.

(H) The growth effects of the Ipl1-Mad2 SPI (and controls) are shown both in wild-type cells and *mad3Δ* cells.

(I) The growth effects (LGR compared to GBP control) of all the Mad2-GBP constructs (WT and mutants) with GFP strains that showed the strongest growth defects (see File S2 for all the data). C-Mad2 is a constitutively closed version of Mad2 (mutation of leucine 7 to alanine) and mad2-TA/VN is constitutively open version of Mad2 that cannot bind C-Mad2 (mutations of threonine 133 and valine 188 to alanines).
