# Supplementary material for: Synthetic Physical Interactions Map Kinetochore-Checkpoint Activation Regions
Source: G3 (Bethesda). 2016 Jun 8;6(8):2531–42. doi: 10.1534/g3.116.031930 (PMC4978906; doi:10.1534/g3.116.031930)
Supplement: Supplemental Material [file supp_g3.116.031930_FigureS3.pdf]

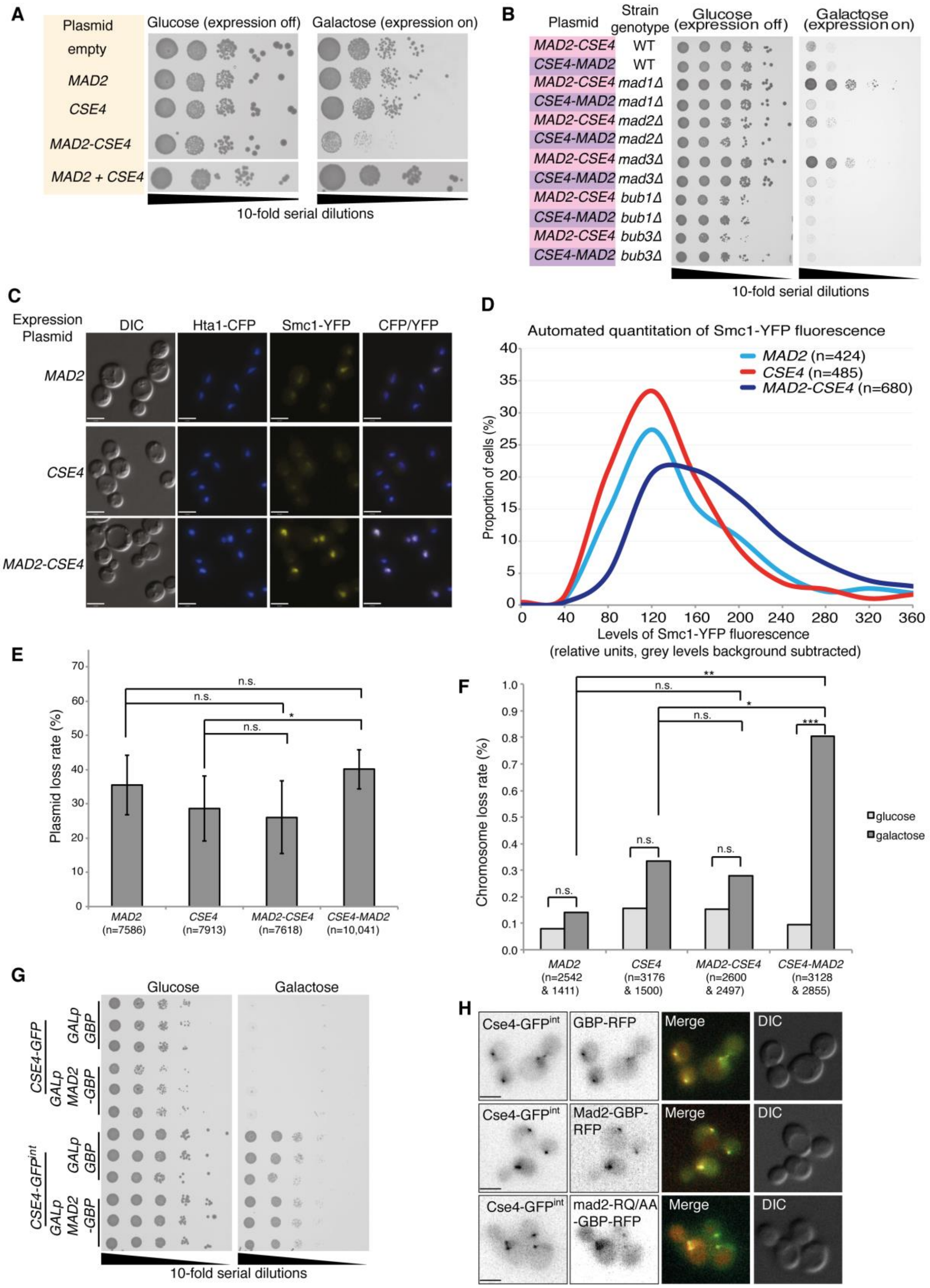

A
Plasmid
Glucose (expression off)
Galactose (expression on)
empty
MAD2
CSE4
MAD2-CSE4
MAD2 + CSE4
10-fold serial dilutions
B
Plasmid
Strain genotype
Glucose (expression off)
Galactose (expression on)
MAD2-CSE4 WT
CSE4-MAD2 WT
MAD2-CSE4 mad1Δ
CSE4-MAD2 mad1Δ
MAD2-CSE4 mad2Δ
CSE4-MAD2 mad2Δ
MAD2-CSE4 mad3Δ
CSE4-MAD2 mad3Δ
MAD2-CSE4 bub1Δ
CSE4-MAD2 bub1Δ
MAD2-CSE4 bub3Δ
CSE4-MAD2 bub3Δ
10-fold serial dilutions
C
Expression Plasmid
DIC
Hta1-CFP
Smc1-YFP
CFP/YFP
MAD2
CSE4
MAD2-CSE4
D
Automated quantitation of Smc1-YFP fluorescence
MAD2 (n=424)
CSE4 (n=485)
MAD2-CSE4 (n=680)
Proportion of cells (%)
Levels of Smc1-YFP fluorescence
(relative units, grey levels background subtracted)
E
Plasmid loss rate (%)
n.s.
*
MAD2 (n=7586)
CSE4 (n=7913)
MAD2-CSE4 (n=7618)
CSE4-MAD2 (n=10,041)
F
Chromosome loss rate (%)
**
n.s.
*
***
glucose
galactose
MAD2 (n=2542 & 1411)
CSE4 (n=3176 & 1500)
MAD2-CSE4 (n=2600 & 2497)
CSE4-MAD2 (n=3128 & 2855)
G
Glucose
Galactose
CSE4-GFP
CSE4-GFP^int
GALp GBP
GALp MAD2 -GBP
10-fold serial dilutions
H
Cse4-GFP^int
GBP-RFP
Mad2-GBP-RFP
mad2-RQ/AA -GBP-RFP
Merge
DIC

**Figure S3 Analysis of *MAD2-CSE4* direct fusion and Mad2-Cse4 SPI.**

(A) Cells were transformed with plasmids expressing nothing (empty plasmid), *MAD2*, *CSE4* or a direct *MAD2-CSE4* fusion, as well as cells expressing both *MAD2* and *CSE4* together, and plated on glucose and galactose. The latter condition drives expression of the transgene and only the *MAD2-CSE4* fusion restricts growth.

(B) Six strains, wild-type (WT), *mad1Δ*, *mad2Δ*, *mad3Δ* , *bub1Δ* and *bub3Δ*, were transformed with either the *MAD2-CSE4* fusion, as in (A), or the reverse *CSE4-MAD2* fusion. Serial dilution assay shows both fusions inhibit growth when expressed (galactose), but only the *MAD2-CSE4* fusion is suppressed in *mad1Δ* and *mad3Δ* strains.

(C) Expression of *MAD2-CSE4* stabilizes cohesion as shown by fluorescence images of cells encoding tagged histone (Hta1-CFP) and cohesin (Smc1-YFP) expressing *MAD2*, *CSE4* or the *MAD2-CSE4* fusion.

(D) An automated image analysis protocol was used to measure the Smc1-YFP fluorescence using the Hta1-CFP fluorescence as a guide for the nuclear volume. The *MAD2-CSE4* fusion elevates the levels of Smc1 fluorescence compared with controls.

(E) A plasmid-loss assay was performed with *mad3Δ* strains containing the *MAD2*, *CSE4, MAD2-CSE4* and *CSE4-MAD2* plasmids along with an empty plasmid with a different selection marker (NAT). The expression of the *MAD2-CSE4* or *CSE4-MAD2* fusions did not significantly increase plasmid loss rate (of the empty NAT plasmid) compared to the expression of *MAD2* alone. There was a slight but detectable (* = *t*-test *p*-value of 0.015) increase in plasmid loss when expressing *CSE4-MAD2* fusion compared to *CSE4* alone, but no difference when the *MAD2-CSE4*

fusion was expressed compared with *CSE4*. Error bars indicate standard deviation of the median and n.s. indicates no statistical difference.

(F) Chromosome-loss assay was performed to assess if cells expressing *MAD2-CSE4* fusions have an increased chromosome-loss rate. Expression of *MAD2* fused to the N terminus of *CSE4* did not increase chromosome loss compared to controls. In contrast, the expression of *MAD2* fused to C terminus of *CSE4* results in significantly increased chromosome-loss rate (*** = Fishers exact test $p$-value = $1.6 \times 10^{-5}$, ** = $p$-value = 0.004, * = $p$-value = 0.04). n.s. indicates no statistical difference.

(G) Cells encoding either C-terminal or internal GFP-tagged Cse4 were transformed with plasmids with GBP or *MAD2-GBP* and serial dilutions were plated on glucose and galactose. Expression of both GBP and *MAD2-GBP* arrested growth of cells encoding Cse4 with C-terminal GFP tag, but not internal GFP tag.

(H) Images of cells encoding Cse4 with internal GFP tag show that Mad2-GBP, and the mutant control, are colocalized with Cse4. Scale bars are 5 µm.
