# Supplementary material for: Synthetic Physical Interactions Map Kinetochore-Checkpoint Activation Regions
Source: G3 (Bethesda). 2016 Jun 8;6(8):2531–42. doi: 10.1534/g3.116.031930 (PMC4978906; doi:10.1534/g3.116.031930)
Supplement: Supplemental Material [file supp_g3.116.031930_FigureS4.pdf]

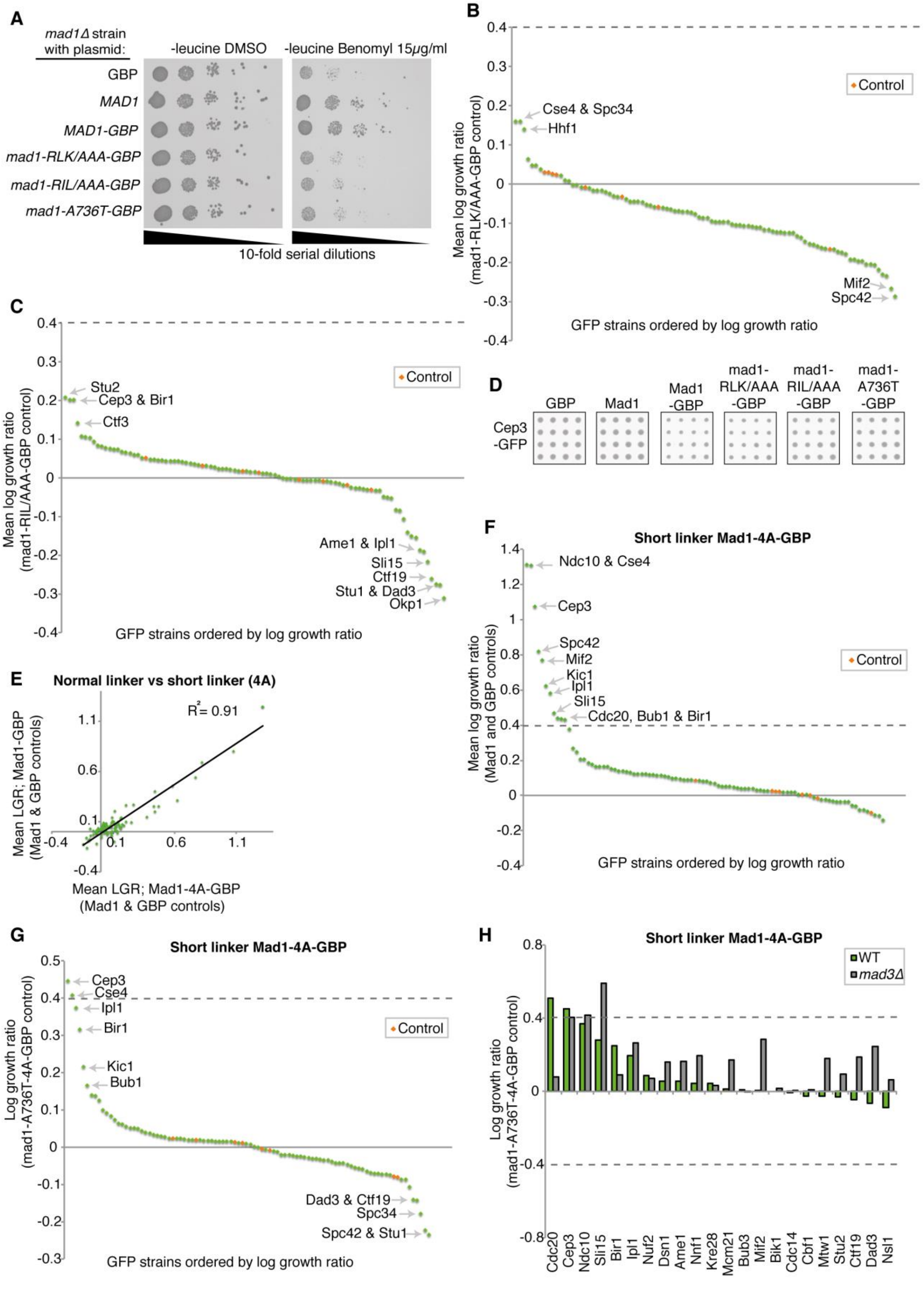
A
mad1Δ strain with plasmid:
-leucine DMSO
-leucine Benomyl 15μg/ml
GBP
MAD1
MAD1-GBP
mad1-RLK/AAA-GBP
mad1-RIL/AAA-GBP
mad1-A736T-GBP
10-fold serial dilutions
B
Mean log growth ratio (mad1-RLK/AAA-GBP control)
Control
Cse4 & Spc34
Hhf1
Mif2
Spc42
GFP strains ordered by log growth ratio
C
Mean log growth ratio (mad1-RIL/AAA-GBP control)
Control
Stu2
Cep3 & Bir1
Ctf3
Ame1 & Ipl1
Sli15
Ctf19
Stu1 & Dad3
Okp1
GFP strains ordered by log growth ratio
D
GBP
Mad1
Mad1 -GBP
mad1- RLK/AAA -GBP
mad1- RIL/AAA -GBP
mad1- A736T -GBP
Cep3 -GFP
E
Normal linker vs short linker (4A)
R² = 0.91
Mean LGR; Mad1-GBP (Mad1 & GBP controls)
Mean LGR; Mad1-4A-GBP (Mad1 & GBP controls)
F
Short linker Mad1-4A-GBP
Mean log growth ratio (Mad1 and GBP controls)
Control
Ndc10 & Cse4
Cep3
Spc42
Mif2
Kic1
Ipl1
Sli15
Cdc20, Bub1 & Bir1
GFP strains ordered by log growth ratio
G
Short linker Mad1-4A-GBP
Log growth ratio (mad1-A736T-4A-GBP control)
Control
Cep3
Cse4
Ipl1
Bir1
Kic1
Bub1
Dad3 & Ctf19
Spc34
Spc42 & Stu1
GFP strains ordered by log growth ratio
H
Short linker Mad1-4A-GBP
Log growth ratio (mad1-A736T-4A-GBP control)
WT
mad3Δ
Cdc20
Cep3
Ndc10
Sli15
Bir1
Ipl1
Nuf2
Dsn1
Ame1
Nnf1
Kre28
Mcm21
Bub3
Mif2
Bik1
Cdc14
Cbf1
Mtw1
Stu2
Ctf19
Dad3
Nsl1

Figure S4 Mad1 kinetochore SPIs.

(A) Serial dilutions of *mad1Δ* strain shows that cells with plasmids containing *MAD1* or *MAD1-GBP* rescue benomyl sensitivity of *mad1Δ*, but not plasmids containing GBP, *mad1-RLK/AAA-GBP, mad1-RIL/AAA-GBP* or *mad1-A736T-GBP*.

(B-C) A set of 88 kinetochore and kinetochore-associated GFP-tagged proteins (Table S1) were tested with Mad1-GBP. Mad1-GBP is compared with mad1-RLK/AAA-GBP (B) or mad1-RIL/AAA-GBP (C).

(D) The growth effects of the Cep3-Mad1 SPI (and controls) are shown.

(E-F) The same 88 GFP strains were screened with a variant of Mad1-GBP with a shorter linker (four amino acids instead of the normal eight), Mad1-4A-GBP, and compared with GBP and Mad1 controls and produce similar results as for the longer linker (Figure 3A) (squared correlation coefficient, R^2=0.91).

(G) The same GFP strains were screened with a version of Mad1-GBP with the shorter linker, Mad1-4A-GBP and compared with mad1-A736T-4A –GBP control. (H) The *MAD3* gene was deleted from 22 GFP-tagged kinetochore strains and the WT and *mad3Δ* strains were retested for their sensitivity to Mad1-4A-GBP (mad1-A736-4A-GBP used as control). The dashed line indicates a mean LGR of 0.4 and the control strains are untagged BY4741. All data for these Mad1 screens are listed in File S3.
