# Supplementary material for: Synthetic Physical Interactions Map Kinetochore-Checkpoint Activation Regions
Source: G3 (Bethesda). 2016 Jun 8;6(8):2531–42. doi: 10.1534/g3.116.031930 (PMC4978906; doi:10.1534/g3.116.031930)
Supplement: Supplemental Material [file supp_g3.116.031930_FigureS5.pdf]

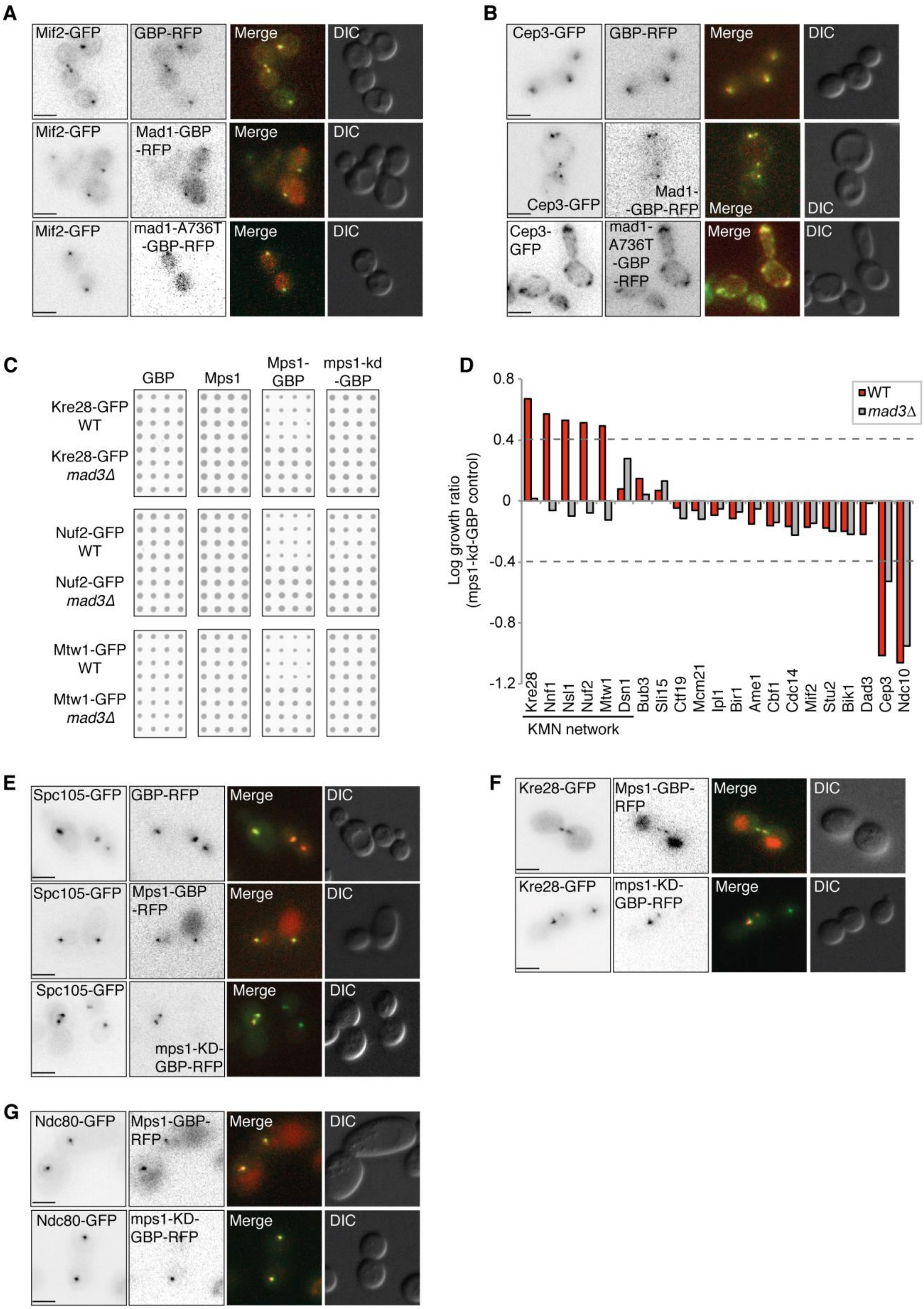

A
Mif2-GFP
GBP-RFP
Merge
DIC
Mad1-GBP-RFP
mad1-A736T-GBP-RFP
B
Cep3-GFP
GBP-RFP
Merge
DIC
Mad1--GBP-RFP
mad1-A736T-GBP-RFP
C
GBP
Mps1
Mps1-GBP
mps1-kd-GBP
Kre28-GFP WT
Kre28-GFP mad3Δ
Nuf2-GFP WT
Nuf2-GFP mad3Δ
Mtw1-GFP WT
Mtw1-GFP mad3Δ
D
WT
mad3Δ
Log growth ratio (mps1-kd-GBP control)
0.8
0.4
0
-0.4
-0.8
-1.2
Kre28
Nnf1
Nsl1
Nuf2
Mtw1
Dsn1
Bub3
Sli15
Ctf19
Mcm21
Ipl1
Bir1
Ame1
Cbf1
Cdc14
Mif2
Stu2
Bik1
Dad3
Cep3
Ndc10
KMN network
E
Spc105-GFP
GBP-RFP
Merge
DIC
Mps1-GBP-RFP
mps1-KD-GBP-RFP
F
Kre28-GFP
Mps1-GBP-RFP
Merge
DIC
mps1-KD-GBP-RFP
G
Ndc80-GFP
Mps1-GBP-RFP
Merge
DIC
mps1-KD-GBP-RFP

Figure S5 Further Mad1 and Mps1 kinetochore SPI analysis.

(A-B) Mad1-GBP and mad1-A736T-GBP are both recruited to (A) Mif2-GFP and (B) Cep3-GFP, the latter of which shows unusual localization when associated with either Mad1 or its mutant version.

(C) The growth effects of the Mps1-KMN network SPIs (and controls) are shown both in wild-type and *mad3Δ* cells.

(D) We deleted the *MAD3* gene in 21 GFP-tagged kinetochore strains and repeated the assay with Mps1-GBP compared with mps1-kd-GBP. The KMN-Mps1 interactions are all suppressed by deletion of *MAD3*.

(E-G) Fluorescence imaging of cells encoding Spc105-GFP (E), Kre28-GFP (F) and Ndc80-GFP (G), shows Mps1-GBP and mps1-kd-GBP are colocalized with the GFP-tagged kinetochore protein. Fluorescence contrast differs between images and the scale bar is 5 µm.
